# Non-inversion conservation tillage as an underestimated driver of tillage erosion

**DOI:** 10.1038/s41598-022-24749-7

**Published:** 2022-12-01

**Authors:** L. K. Öttl, F. Wilken, A. Hupfer, M. Sommer, P. Fiener

**Affiliations:** 1 Institute of Geography, University of Augsburg, Alter Postweg 118, 86159 Augsburg, Germany; 2 Landscape Pedology Working Group, Leibniz Center for Agricultural Landscape Research ZALF e.V., Eberswalder Str. 84, 15374 Müncheberg, Germany; 3 Institute of Environmental Science and Geography, University of Potsdam, Karl-Liebknecht-Str. 24-25, 14476 Potsdam, Germany

**Keywords:** Ecology, Environmental sciences

## Abstract

Tillage erosion is a widely underestimated process initiating soil degradation especially in case of large agricultural fields located in rolling topography. It is often assumed that, conservation, non-inversion tillage causes less tillage erosion than conventional inversion tillage. In this study, tillage erosion was determined on three paired plots comparing non-inversion chisel versus inversion mouldboard tillage. The experiments were performed at three sites in Northeast Germany with gentle, moderate, and steep slope, while tillage depth (0.25 m) and speed (≈ 6 km h^−1^) were kept constant during all experiments. The results indicate that non-inversion tillage produces significantly more soil movement compared to inversion tillage. The soil translocation distance was by a factor of 1.3–2.1 larger in case of chisel tillage. The largest difference in translocation distance and tillage transport coefficient (*k*_*til*_) was found on the gentle slope exhibiting the lowest soil cohesion. Our results together with an evaluation of *k*_*til*_ values derived from literature and standardised for 0.25 m tillage depth contradict the general assumption that non-inversion tillage reduces tillage erosion. In tillage erosion dominated areas, non-inversion tillage applied with high tillage speed and depth potentially increases tillage erosion and fails its purpose to serve as soil conservation measure.

## Introduction

Soil erosion is a major threat for world’s soils^1,2^ that critically endangeres the supply of soil ecosystem services such as food production, biodiversity, carbon storage and water quality^3^. Soil erosion due to water and wind occurs in natural and human-dominated environments, where especially arable management increases erosion processes due to prolonged times of bare soil following tillage operations. One very effective way of reducing soil erosion on arable land is to reduce tillage intensity and improve residue or mulch cover on soil surfaces^4–6^. Typically, this is done via non-inversion mulch tillage (conservation tillage) or direct seeding without tillage (no-till) systems^7^. At least in Europe no-till does not play a big role, while conservation tillage is increasingly applied due to economic (saving costs of labour and machinery) and ecological benefits^8^ (e.g. in Germany: at 1% and 37% of the arable land no-till and conservation tillage are applied, respectively^9^).

On arable land, another important but less recognised erosion process is tillage erosion, causing substantial down-slope movement of soil. On global scale, it is estimated that tillage erosion equates a fifth of water erosion and twice as much as wind erosion^10^. In regions with limited erosive rainfall, tillage erosion can be the dominant soil degradation process (e.g. in Northeast Germany^11,12^), which takes place wherever soils are tilled on sloped land regardless of climatic conditions. In addition, progressive mechanisation of agriculture since the mid of the twentieth century leads to increasing tillage erosion rates^12–14^. Tillage erosion is related to slope gradient, where changes in gradient either lead to local soil loss or gain. Furthermore, tillage erosion is driven by the kind of tillage implement (type, shape, and tool size), operational conditions (tillage depth, speed, and direction), field parameters (field size and boundaries), and soil properties (soil texture, soil moisture, and bulk density)^13^.

As tillage erosion does not lead to off-site effects causing obvious damage in surrounding ecosystems by sediment deposition (along streets, in-streams, etc.) as it is the case for water and wind erosion, the latter receive much higher attention. Determining tillage erosion requires different measuring techniques compared to water and wind erosion, where sediment can be trapped at the ‘outlet’ of an area under study^15^. Assessing tillage erosion can be based on different monitoring techniques such as topographic change^16–18^ or tracers. These tracers are either added before performing individual or a series of tillage operations^19–22^ or in-situ tracers, e.g. fallout radionuclides^12,23,24^, are used to estimate long-term erosion rates, which in the latter case account for all erosion processes. An overview and comparison of methods for assessing tillage erosion is given in Fiener et al.^16^.

Compared to water and wind erosion there are hardly any targeted measures to reduce or avoid tillage erosion. No-till practice keeps the soil structure intact and causes minimum soil disruption and translocation^25^, and is an effective measure combating water, wind and tillage erosion. However, for much more frequently applied non-inversion tillage, it is not clear if this practice has a reducing effect on tillage erosion. Overall, few studies assessed tillage erosion driven by non-inversion tillage compared to inversion tillage^19,26–29^. Analysing the published differences in tillage erosion due to inversion tillage and non-inversion tillage indicates that the latter (mostly based on different chisel ploughs) tends to induce smaller erosion rates^26,28,30^. However, the smaller tillage erosion rate seemed to be often associated with smaller tillage depths in case of chisel plough systems compared to traditional mouldboard ploughing^13^. Moreover, it is important to note that there are also few studies^19,29^ indicating that non-inversion tillage has even higher tillage erosion rates as compared to inversion tillage, which might be related to higher tillage speeds that are sometimes applied to non-inversion implements^19,31^.

The aim of this study is to determine differences in tillage erosion intensity between a non-inversion chisel plough and an inversion mouldboard plough on different paired slopes, while keeping tillage speed and depth constant to ensure comparability. It is hypothesised that for the same tillage speed and depth, inversion and non-inversion tillage cause similar tillage erosion rates.

## Materials and methods

### Research area and experimental sites

The research area is the “AgroScapeLab Quillow” located approximately 100 km north of Berlin, Germany. It represents a typical ground moraine landscape formed after the retreat of the Weichselian glaciers (ca. 15 ka BP) in Northeast Germany^32^. The hummocky area is characterized by a hilly topography with short summit-footslope distances (on average 35 m). Due to its undulating topography (mean slope ca. 7% ± 6%; 74% of the area with a slope > 3%), large field sizes (mean field size 13 ha ± 18 ha; 2–150 ha) and highly mechanized arable farming, the region faces severe soil degradation by tillage erosion^11,12^. Generally, extremely eroded A-C profiles (Calcaric Regosols) occur at convex knolls and steep slopes. Strongly eroded soils (Nudiargic Luvisols) cover upper slopes and non-eroded soils (Calcic Luvisols) dominate at lower midslopes. Footslope areas and closed depressions show colluvial soils (Colluvic Regosols), often influenced by near-surface groundwater (for illustration of soil profiles please refer to^11,12^). Overall, the spatial distribution of soil types is closely linked to soil redistribution processes and terrain position^33–35^. Soil texture of Ap horizons in the region ranges from loamy sand to sandy clay loam, depending on soils’ erosion status. The climate is subcontinental with an average annual air temperature of 9.4 °C and a mean annual precipitation of 466 mm (20-year average 2001–2020, DWD meteorological station at Grünow^36,37^).

**Figure 1:**
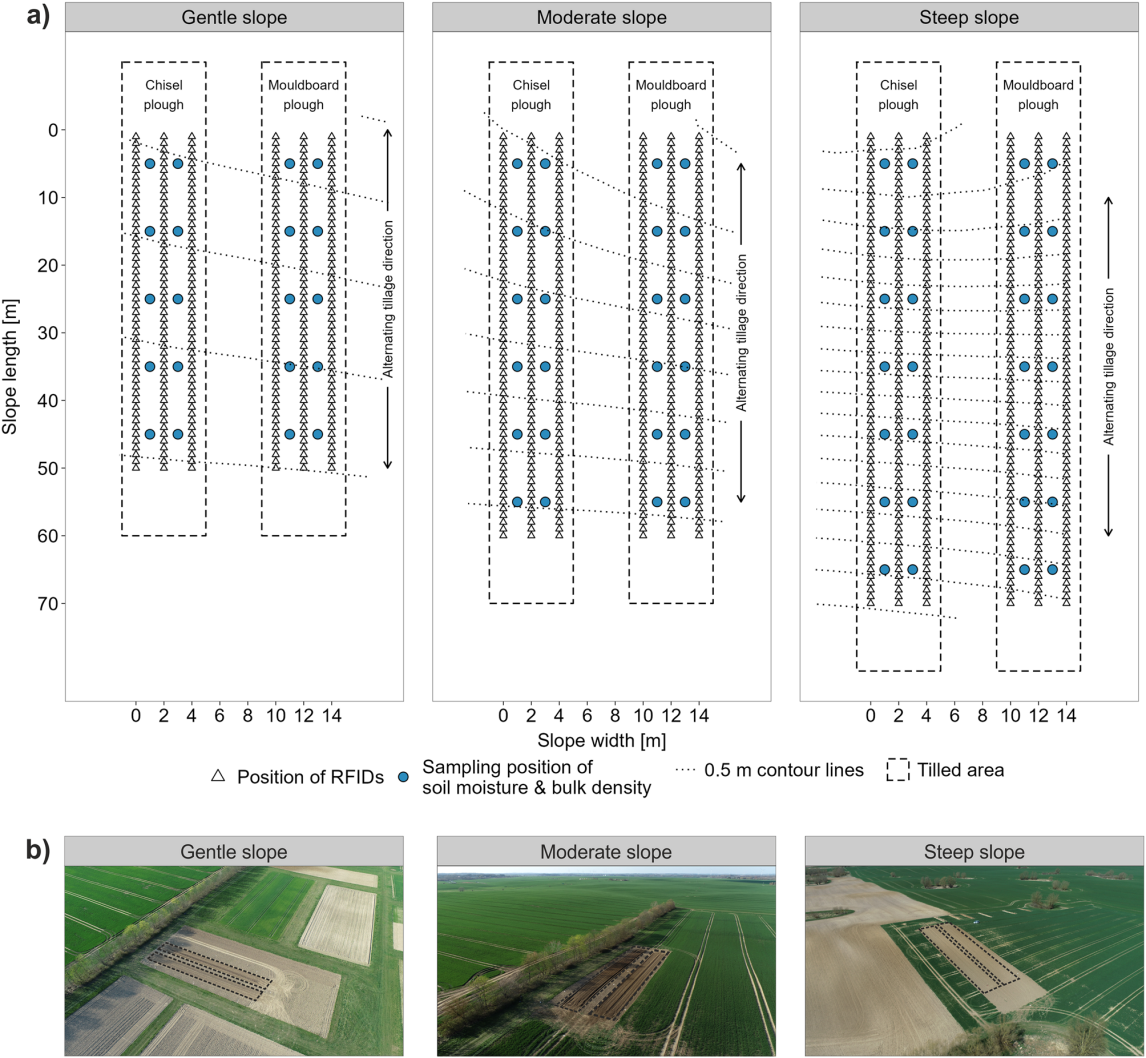
(**a**) Experimental design of the tillage experiments. Separate plots for chisel plough (left) and mouldboard plough (right) tillage next to each other at each of the three experimental sites with gentle, moderate, and steep slope. Three rows of RFIDs (triangles) for each plot with 2 m distance between the rows and 1 m between the RFIDs along the slope. Soil moisture and bulk density were measured in lines between the RFID rows starting after 5 m with 10 m increments (blue dots). Dotted lines indicate contour lines (0.5 m interval). Dashed boxes mark the tilled area, whereby tillage direction was alternating up- and down-slope. Please note the different plot lengths per site. (**b**) Aerial photos of the experimental sites (black dashed boxes) that are located at 53.370546° N 13.800004° E (gentle slope), 53.374694° N 13.799799° E (moderate slope), and 53.421454° N 13.678403° E (steep slope).

Tillage experiments were performed at three experimental sites managed by the research station of the Leibniz Center for Agricultural Landscape Research (ZALF) in Dedelow (federal state of Brandenburg, Northeast Germany). The sites were selected following a topographic gradient with slopes of 3.5%, 5.9%, and 11.8% (Fig. 1), which in the following are referred to as gentle, moderate, and steep slope (*GeS*, *MoS*, and *StS*, respectively). Compared to *GeS* and *MoS*, the steepest slope *StS* showed a somewhat more variable soil texture following topography and erosion status. Overall, the topsoils of the *GeS* have a coarser texture (d_50_ = 0.093 mm; 64% sand, 29% silt, 7% clay) than those of the *MoS* (d_50_ = 0.077 mm; 57% sand, 30% silt, 13% clay) and *StS* (d_50_ = 0.079 mm; 55% sand, 29% silt, 17% clay).

### Experimental design

The three experimental sites were subdivided in two paired plots with a width of 4 m each and equipped with tracers over a slope length of 50, 60, and 70 m at the *GeS*, *MoS*, and *StS*, respectively (Fig. 1). To avoid cross-contamination with tracers between the plots, a buffer of 5 m was established between them. Radiofrequency identification transponder glass tags (RFIDs; Smartrac, Avery Dennison, US) with a frequency of 125 kHz, a diameter of 0.4 cm, a length of 2.2 cm, and a density of 2.3–2.5 g cm^−3^ were placed regularly within the plots (Fig. 1). The RFIDs were inserted in three rows per plot with a spacing of 2 m between the rows and 1 m between the RFIDs along the slope in a depth of 0.125 m (half of ploughing depth). This resulted in 150, 180, and 210 RFIDs per plot on the *GeS*, *MoS*, and *StS*, respectively.

**Figure 2:**
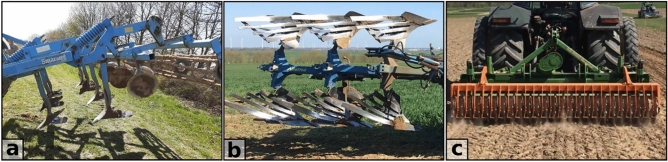
Implements used within tillage experiments: (**a**) chisel plough, (**b**) mouldboard plough, and (**c**) roller.

The experiment was carried out during the typical time of tillage in the region end of April 2021. For homogenous starting conditions, all three experimental slopes were prepared with a chisel plough (tillage depth 0.2 m). Tillage experiments on the paired plots were performed with a chisel and a mouldboard plough representing soil conserving, non-inversion and conventional, inversion tillage, respectively, whereby both tillage implements were always followed by a roller (Fig. 2). Tillage depth was chosen to be 0.25 m for both implements as this is a typical tillage depth in the study area. Both implements tilled alternating five times up- and down-slope per plot (10 times in total). The translocation distance was retrieved from the difference in the coordinates and calculated for left, right, up- and down-slope direction and the resulting net distance. Results are given in translocation distance per pass, i.e. the measured translocation distance divided by ten.

The compared implements utilised during the experiment are a chisel and mouldboard plough that were operated by tractors of 150 hp. The wing-shared chisel plough (Smaragd, Lemken, Germany; Fig. 2a) consists of seven duck feet followed by six discs for crumbling soil clods and a cage roller for re-compaction of the soil. The implement has a working width of 3 m and operated at a tillage speed of 6.4–7.0 km h^−1^. It took two passes next to each other in one direction to cover the full plot width of 4 m. The three-bladed mouldboard plough (Albatros, Raabe, Germany; Fig. 2b) has a working width of 1.5 m and was operated at a comparable tillage speed of 6.3–6.5 km h^−1^. It took four passes per direction to cover the full plot width of 4 m. After each complete up- or downward tillage pass over the full plot width a tooth packer roller (Amazone, Germany; Fig. 2c) was applied for soil re-compaction (Fig. 2c).

### Determining soil properties and soil movement

Soil moisture and bulk density were measured in a regular grid at each of the six plots (Fig. 1). Soil moisture was measured using a hand-held FDR (frequency domain reflectometry) soil moisture probe (ThetaProbe ML3 Delta-T Devices, UK) shortly before the tillage experiments started. At each measurement position, nine single measurements were taken and averaged. Soil samples for determining bulk density were taken with a liner sampler (set B, Eijkelkamp, Netherlands) that takes an undisturbed soil core of 0.037 m diameter and 0.2 m length. At each measurement position (Fig. 1), a mixed soil sample of two samples was taken before and after the experiments. Before weighing the soil samples, they were oven dried at 105 °C for at least 60 h.

**Figure 3:**
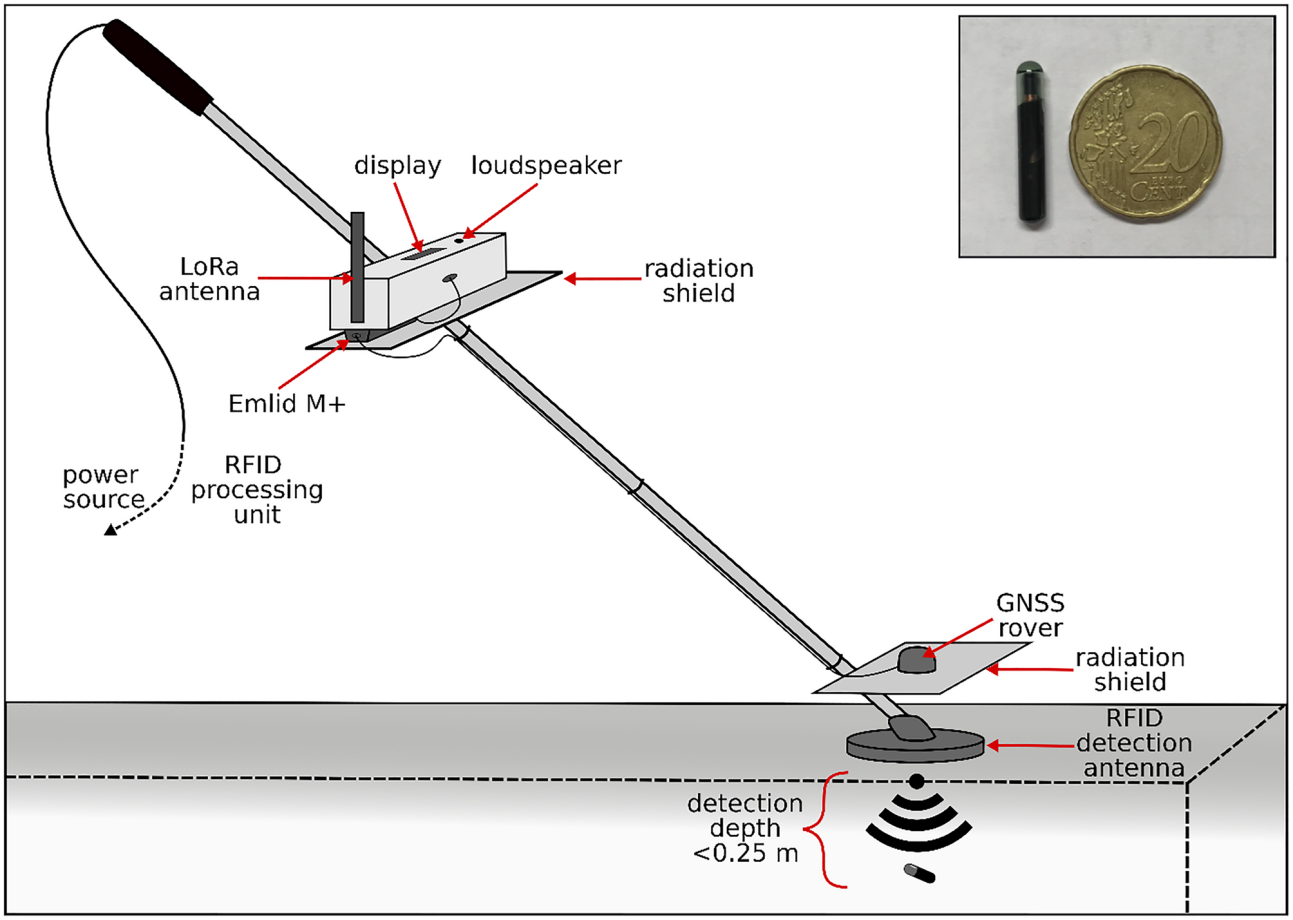
Schematic drawing of the RTK GNSS RFID (real time kinetic global navigation satellite system radio-frequency identification) detection and geolocation system. The lower part (direct proximity to soil surface) of the setup consists of an RFID and GNSS antenna, which are located above each other. The upper part consists of the processing unit for RFID identification and RTK GNSS module that communicates with a geostationary base station (not shown) via long-range radio (LoRa) to receive correction data. The inset frame contains a photo of an RFID tag next to a coin acting as scale.

Movement of RFID tags was measured with a detection antenna (Fig. 3, Rolling Stone, TECTUS, Germany) with a diameter of 0.125 m and a soil penetration depth between 0.20 and 0.25 m. The attached RFID reader indicates a detected transponder via a sound signal and logs the ID number of the detected RFID together with detection time and coordinates. The location of the detected RFIDs is determined using RTK GNSS (real time kinetics global navigation satellite system) correction. A geostationary base station (Reach RS +, Emlid, China) was set-up over fixed reference points at each slope. The base station sent real-time correction to the GNSS rover (Reach M +, Emlid, China; satellite constellation GPS and GALILEO, frequency 5 Hz) of the RFID detection system to achieve accuracies of about 0.05 m^38^. The uncertainty of the RFID position obtained by the GPS measurements was estimated via two approaches. One approach was to insert four RFID transponders per site at locations that are not affected by translocation during the tillage experiments (grass strips nearby each field corner). The position of those RFIDs was measured together with all other RFIDs before and after the experiments. The second approach compares the RTK GNSS coordinates of the RFID detection system against high accuracy total station measurements (TS06plus, Leica Geosystems AG, Switzerland). This comparison was exemplarily done at *MoS*. The comparison focused on potential geo-rectifications that go back to the RTK GNSS measurements. The major advantage of the RFID detection system is that it can be conducted by only one person alone compared to the use of a total station where at least 2 people are needed.

### Data analysis

Initially, a coordinate transformation from UTM to a local coordinate system was applied where plot width is on the x-axis and plot length on the y-axis. Positive values indicate a translocation in upslope direction and negative values a down-slope movement relative to the starting position of the RFID, respectively.

To calculate the tillage transport coefficient *k*_*til*_ (as used in many models e.g. WaTEM/SEDEM, SPEROS-C) for all plots and tillage implements, the plots where subdivided into 10 m increments along down-slope direction. Subsequently, mean down-slope transport distances ${\overline{d} }_{n}$ per pass were calculated based on RFID translocation within these segments. Based on the assumption that ${\overline{d} }_{n}$ per segment is proportional to slope^13,26,39^, *k*_*til*_ was calculated per segment following Eqs. (1) and (2) according to Govers et al.^26^.

1 $${\overline{d} }_{n}= b {S}_{n}$$

2 $${k}_{til}=D {\rho }_{b} b$$

Thereby, *b* is the linear regression slope, *S*_*n*_ is slope tangent, *D* is tillage depth (0.25 m in the experiments), and ${\rho }_{b}$ is bulk density, whereas the mean bulk density is used for all slope increments per slope.

An unpaired two-sample Wilcoxon rank sum test was performed to compare the mean transport distance $\overline{d }$ and the mean *k*_*til*_ between the plots. Moreover, this test was used to compare translocation directions (up- vs. downslope and up-/downslope vs. left/right) per implement and between the implements. This non-parametric test is an alternative to the unpaired two-sample t-test that is used when data is not normally distributed^40^. All figures showing data are generated with the R package ggplot2^41^ and all analysis were performed in RStudio 2021.09.2 with R version 4.1.2^42^.

## Results

The positional uncertainty of the RFID detection system assessed by the geostationary RFIDs revealed a mean (± one standard deviation) positional error of 0.1 ± 0.2 m, while the mean absolute net translocation distance over all fields was 2.2 ± 2.3 m. A somewhat lower accuracy was shown for the *GeS* (0.17 ± 0.17 m), which is likely caused by disturbance originating from a nearby cell tower. For *MoS* and *StS*, the accuracy was 0.05 ± 0.03 m and 0.05 ± 0.01 m, respectively. At all test slopes the deviation between the repeated measurements of the geostationary RFIDs was randomly distributed in all spatial directions.

The mean recovery rate for all plots after ten tillage passes was 66 ± 11%. In general, the recovery rate of the RFIDs was higher for the chisel plough plots (67%, 73%, and 76% for the *GeS*, *MoS*, and *StS*, respectively) compared to the plots tilled by mouldboard plough (53%, 57%, and 46% for the *GeS*, *MoS*, and *StS*, respectively).

**Figure 4:**
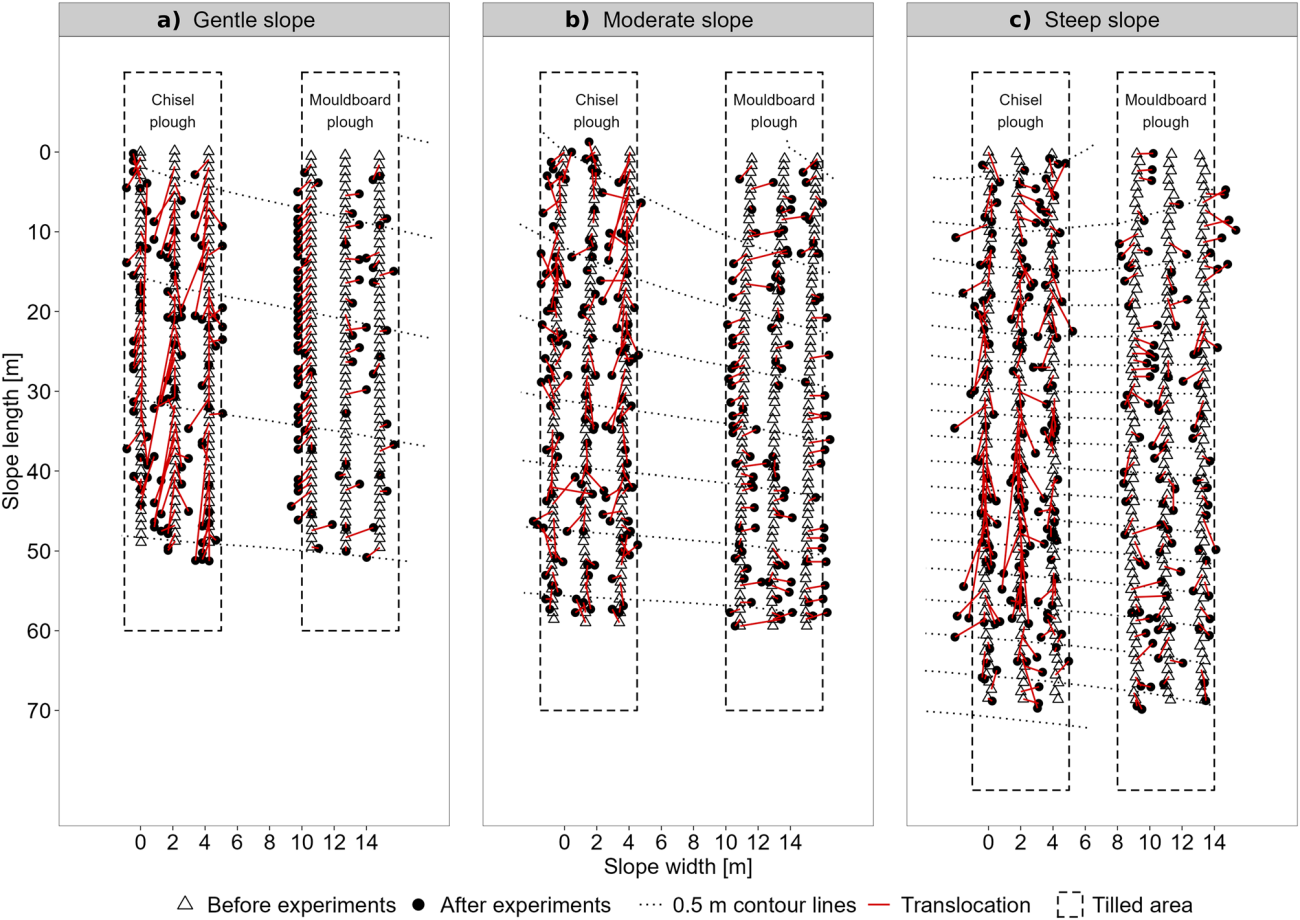
Spatial pattern of the position of the RFIDs before (triangles) and after the experiments (black dots) for the three experimental sites with (**a**) gentle, (**b**) moderate, and (**c**) steep slope. Tillage by chisel plough (left) and mouldboard plough (right), respectively. Red lines indicate the net movement of the individual RFID transponders. Dotted lines indicate contour lines (0.5 m interval) of the digital elevation model and dashed boxes mark the tilled area whereby tillage direction was alternating up- and downslope.

**Figure 5:**
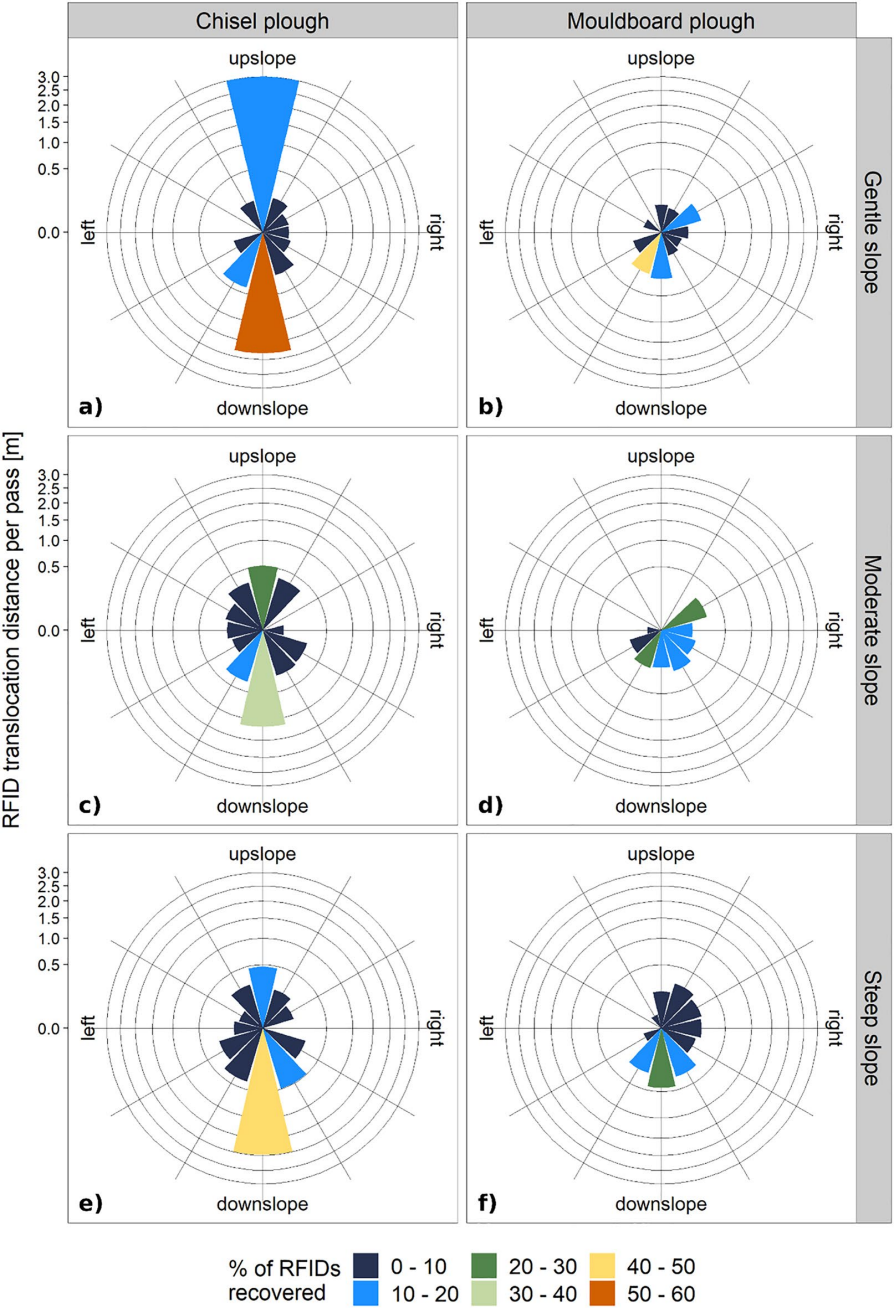
Direction and net translocation distance of the RFID translocation per pass [m] for the two tillage implements chisel and mouldboard plough (in columns) and the three experimental sites (**a**,**b**) gentle, (**c**,**d**) moderate, and (**e**,**f**) steep slope (in rows). Colours indicate the percentage of RFIDs from all inserted RFIDs per experimental plot (**a**–**f**) that were translocated in each direction (360° divided in 12 segments of 30° each). Direction of translocation is related to the field geometry. Please note that the y-axes is square root transformed, i.e. unequally sized space between axis breaks for a better comparison of chisel and mouldboard plough data.

As expected, the dominant tillage translocation is in down-slope direction (p-value < 0.01 for the three test sites, respectively; Figs. 4, 5), whereas in case of the mouldboard plough the movement in tillage direction (up- and downslope) is less pronounced due to a sideward movement during soil inversion (p-value < 0.1 for the three test sites, respectively). For all slopes, the variation in RFID transport distance is much higher for chisel plough compared to mouldboard plough (Fig. 4), which indicates more pronounced soil mixing during tillage operations.

**Figure 6:**
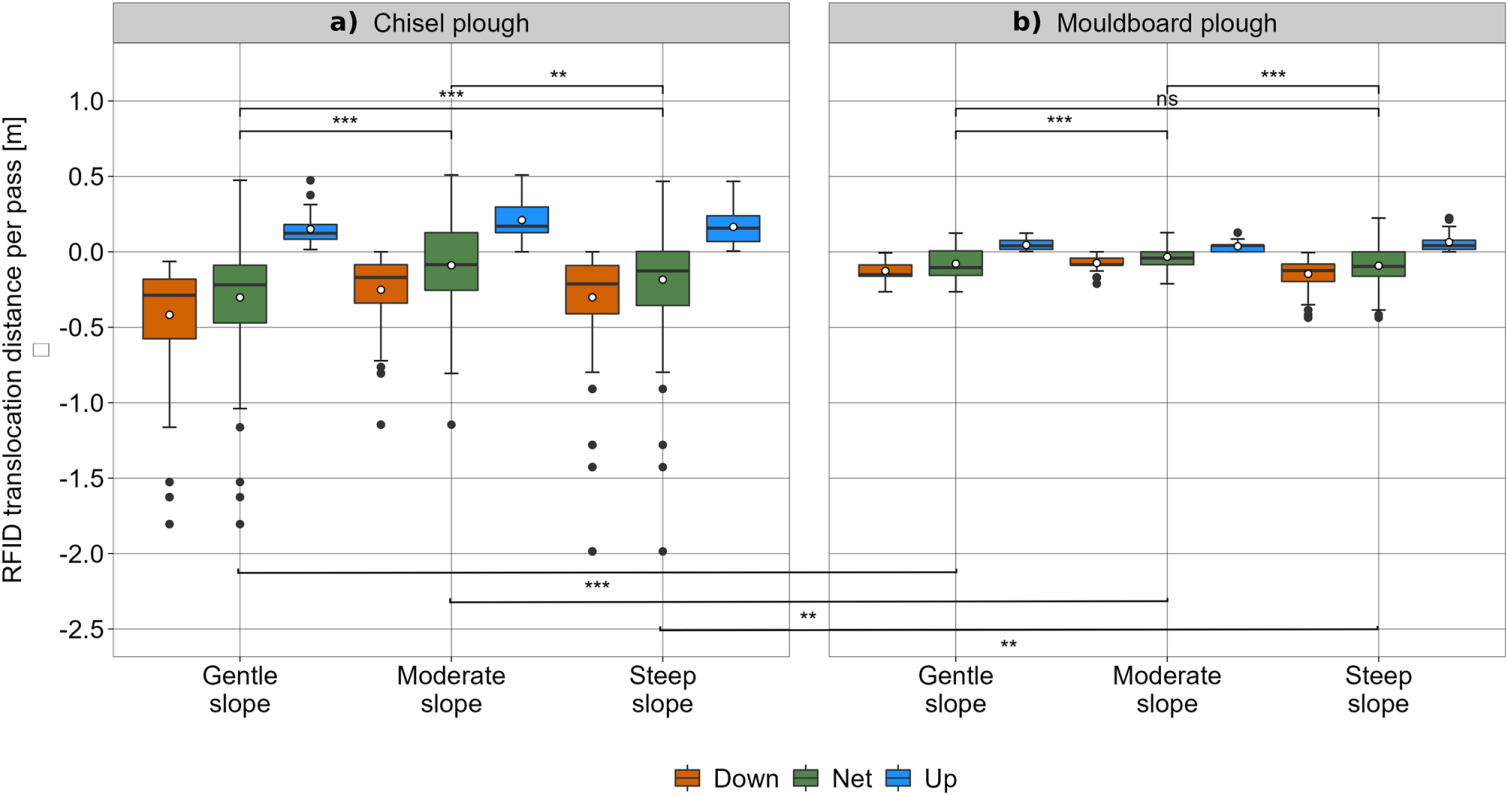
Boxplot of the RFID translocation distance per pass [m] for the three experimental sites with gentle, moderate, and steep slope. Comparison of the tillage implements (**a**) chisel plough and (**b**) mouldboard plough as well as down-slope (orange), up-slope (blue), and net translocation (green) of the RFIDs. Boxes indicate 1st quartile, median and 3rd quartile, whiskers indicate ± 1.5 times the inter-quartile range, while dots represent data beyond the end of the whiskers. White circles indicate mean values per boxplot. Stars denote significance levels of the Wilcoxon rank sum test for difference in means (ns: p-value > 0.05, *: p value < 0.05, **: p value < 0.01, ***: p value < 0.001).

The chisel plough led to a significantly larger mean down-slope soil translocation indicating a more pronounced tillage erosion effect (Fig. 6). Overall, the chisel plough led to a 342%, 270%, and 200% larger mean (207%, 202%, and 131% median) net down-slope soil transport as compared to the mouldboard plough for the paired plots on *GeS*, *MoS*, and *StS*, respectively (Fig. 6). It is interesting to note that differences between chisel plough and mouldboard plough decreased with increasing slope steepness.

**Figure 7:**
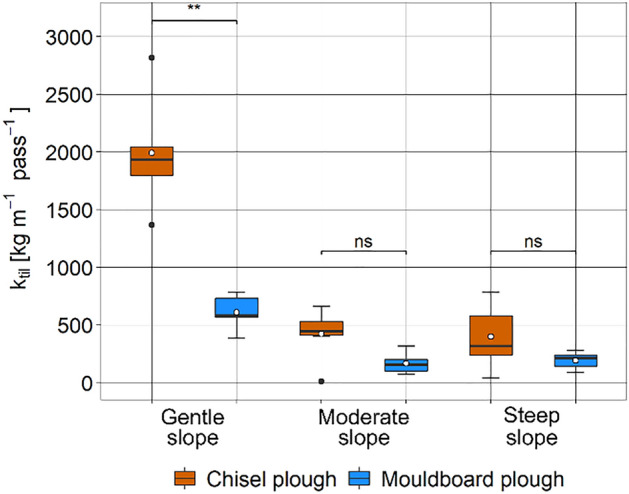
Boxplots of ﻿*k*_til_ [kg m^−1^ pass^−1^] for the three experimental sites with gentle, moderate, and steep slope calculated based on slope segments. Boxes indicate 1st quartile, median and 3rd quartile, whiskers indicate ± 1.5 times the inter-quartile range, while dots represent data beyond the end of the whiskers. White circles indicate mean values per boxplot. Stars denote significance levels of the Wilcoxon rank sum test for difference in means (ns: p-value > 0.05, *: p value < 0.05, **: p value < 0.01, ***: p value < 0.001).

Calculating mean *k*_*til*_ values for the different plots and treatments underlines a substantially higher erosion potential of using a chisel plough compared to a mouldboard plough if tillage depth and speed are kept constant (Fig. 7). As *k*_*til*_ is supposed to be independent from slope (see Eqs. 1 and 2), differences for the same implement with similar tillage speed and depth result from differences in soil properties of the plots. Here it is important to note that sandier and especially drier soils at the *GeS* show a higher *k*_*til*_, which indicates a higher erosion potential, particularly for non-inversion tillage (Table 1).

**Table 1:** Soil moisture measured before starting the tillage experiments and bulk density measured before and after the experiments.

Experimental site	Sampling positions	Soil moisture per experiment [w-%]	Bulk density [kg m^−3^]	
Slope	n	Mean (± standard deviation)	Before experiments	After experiments
Gentle (*GeS*)	10	11.8 ± 1.8	1140 ± 100	1250 ± 70
Moderate (*MoS*)	12	15.8 ± 1.9	1210 ± 90	1260 ± 60
Steep (*StS*)	14	16.3 ± 2.4	1120 ± 100	1160 ± 70

## Discussion

The direct comparison between inversion mouldboard versus non-inversion chisel tillage is subject to some uncertainties. The sensitivity of tillage speed is potentially higher for chisel plough compared to mouldboard plough due to the design and purpose of the implement. While a mouldboard plough inverts soil by cutting and moving soil perpendicular to the tillage direction, e.g. illustrated in^43^, chisel tillage induces soil disruption and mixture by stirring soil up and forming a wave-like soil flux. The height and corresponding translocation distance of this wave is controlled by tillage speed. The sensitivity of chisel tillage to speed is also indicated by Van Muysen et al.^22^, reporting almost a doubling of tillage translocation due to a 20% increase of tillage speed (Table 2). To quantify the sensitivity of single tillage implements to tillage speed, a larger set of experiments including different implements, slopes, speeds and depths would be required. In this study, the up- and down-slope tillage speed and depth was kept constant for both implements at all sites (*GeS*, *MoS*, and *StS*). This enabled to focus solely on differences in tillage implements and slope gradients as well as to avoid artificially high down-slope movement. Nevertheless, at *StS*, a minor reduction of upslope tillage speed (upslope speed 5.5 km h^−1^ vs. mean speed of experiment 5.9 km h^−1^) for mouldboard plough was unavoidable due to power limitations of the pulling machinery. Hence, the down-slope translocation at *StS* for mouldboard plough might be slightly overestimated.

**Table 2:** Comparison of tillage erosion coefficients (*k*_*til*_) for inversion (mouldboard plough) vs. non-inversion (chisel plough) up- and down-slope tillage.

Study		Implement	Speed	Depth	*k*_*til*_	Normalized *k*_*til*_
			[km h^−1^]	[m]	[kg m^−1^ per pass]	
**Direct comparison of implements**						
Govers et al.^26^		Mouldboard	4.50	0.28	234	209
		Chisel	4.50	0.15	111	185
Kietzer^31^		Mouldboard	6.00	0.19	138	182
		Chisel	6.10	0.14	250	446
Lobb et al.^19^		Mouldboard	6.20	0.23	364	396
		Chisel	9.60	0.17	275	404
Marques da Silva et al.^30^		Mouldboard	3.70	0.39	770	494
		Chisel	3.60	0.11	75	170
		Chisel	3.40	0.19	27	36
Mech and Free^28^		Mouldboard	3.60	0.08	24	75
		Chisel	3.60	0.06	13	54
Tiessen et al.^29^		Mouldboard	7.00	0.175	43	62
		Chisel	7.00	0.175	64	92
**No direct comparison of implements**						
De Alba^43^		Mouldboard	4.5	0.24	204	213
Gerontidis et al.^44^		Mouldboard	4.5	0.2	153	191
		Mouldboard	4.5	0.3	383	319
		Mouldboard	4.5	0.4	670	419
Heckrath et al.^45^		Mouldboard	4.9	0.25	200	200
		Mouldboard	6.3	0.26	335	322
Kosmas et al.^46^		Mouldboard	4.5	0.18	63	88
		Mouldboard	4.5	0.25	160	160
Lindstrom et al.^47^		Mouldboard	7.6	0.24	330	344
Lobb et al.^48^		Mouldboard	4.0	0.15	184	307
Quine and Zhang^49^		Mouldboard	5.8	0.22	112	127
Quine et al.^50^		Mouldboard	7.0	0.17	324	476
Revel and Guiresse^51^		Mouldboard	6.5	0.27	263	244
Van Muysen and Govers^52^		Mouldboard	5.0	0.25	224	224
		Mouldboard	5.4	0.21	169	201
Van Muysen et al.^53^		Mouldboard	1.8	0.33	245	186
		Mouldboard	2.7	0.15	70	117
Poesen et al.^54^		Chisel	2.3	0.16	282	441
		Chisel	2.3	0.14	139	248
Quine et al.^55^		Chisel	2.2	0.19	657	864
Van Muysen et al.^22^		Chisel	5.8	0.15	225	375
		Chisel	7.2	0.2	545	681
Van Muysen and Govers^52^		Chisel	6.8	0.069	123	446
**Mouldboard**		Mean	5.0	0.2	246	241
		CV [%]	28	32	74	51
**Chisel**		Mean	5.0	0.1	214	342
		CV [%]	47	30	91	72
**This study**		Mouldboard	5.90	0.25	324	
		Chisel	7.10	0.25	1037	

The normalized *k*﻿_*til*_ is calculated for a tillage depth of 0.25 m.

However, it is important to note that the speed of chisel tillage was lower compared to typical speeds applied in the region (approx. 10 km h^−1^ for mouldboard and 12 km h^−1^ for the chisel tillage with commonly used big tractors; information from G. Verch, head of the research station). Hence, the differences between inversion and non-inversion tillage found in this study are rather conservative.

Based on the methodological comparison study by Fiener et al.^16^ it was demonstrated that RFID-based transport tracing is in agreement with established approaches based on different tracers (magnetic iron oxide, fluorescent sand, and RFIDs) and topographic change approaches (terrestrial laser scanning, unmanned aerial vehicle-based structure from motion approaches, and changes in soil depths over buried concrete flagstones). The RFIDs showed a similar transport behaviour compared to other macro-tracers like coloured stones^29^ or metal cubes^52^ used in several earlier studies determining tillage erosion. Hence, in general, the RFID approach is assumed to be suitable to determine soil movement.

The RFID detection system used in this study yielded similar recovery rates as shown in Fiener et al.^16^ for chisel plough (this study: 67–76%; Fiener et al.^16^: 75–79%). It is assumed that the somewhat lower recovery rates in our study are a result of a higher tillage depth, which is close to the detection limit of the antenna (penetrating between 0.20 and 0.25 m into the soil). One could speculate that this leads to a slight overestimation of transport distances as deeper layers of tilled soil horizons might be transported less, while RFIDs moving in these layers are more difficult to locate. However, Fiener et al.^16^ demonstrated that chisel tillage resulted in a mostly homogenous soil mixture within the plough layer based on fluorescent sand.

The mean positional error of the RFID detection system (0.1 m) is an order of magnitude smaller compared to the mean net translocation distance after 10 tillage passes (1.25 m). Although the measured RFID position error did not show any direction, it would result only in a 6.5% reduction of translocation distances or 14% reduction of *k*_*til*_, in case the highest error measured on *GeS* (mean position error = 0.17 m) would have been exclusively occurred in slope direction. However, for translocation assessments of individual tillage passes, the positional accuracy of the RFID detection system might not be sufficient and the use of a total station for RFID positioning is more appropriate.

Regarding the comparison of the two tillage implements, the hypothesis is falsified that non-inversion chisel plough results in similar tillage erosion as mouldboard ploughing as long as tillage depth and speed are kept constant. This study highlights that tillage erosion by non-inversion chisel tillage substantially exceeds conventional, inversion mouldboard tillage practices by a factor of 1.3–2.1 regarding soil erosion under similar tillage depth and speed. Site specific differences for *GeS*, *MoS,* and *StS* are even higher when *k*_*til*_ values are compared (factor 2.9–3.5; Fig. 7). Although the differences in tillage erosion between the implements are not significant at *MoS* and *StS*, especially the difference on the flattest slope (*GeS)* is astonishing (mean net translocation distance of − 0.27 m for chisel and − 0.08 m for mouldboard tillage). Comparing the *k*_*til*_ values with literature data shows that *k*_*til*_ derived for chisel plough is approximately 1.1 times larger as the highest reported values for comparable implements (864 kg m^−1^ per pass in Quine et al.^55^, normalised for 0.25 m tillage depth). The equations used to calculate *k*_*til*_ (Eqs. 1, 2) assume a linear relation between slope and transport distance in case of up- and down-slope tillage^26,39^. However, as the measured transport distances in case of chisel and mouldboard plough on the *GeS* are as high as on the *StS*, they result in very high *k*_*til*_ values for the *GeS* due to the small slope. In addition, the high translocation distances at the *GeS* are assumed to be driven by weak soil cohesion associated with sandy and dry soils^56^ during the experiment (Table 1). However, the effect of soil texture and soil moisture could not be quantified based on the experimental set-up of this study. Nevertheless, our results point at a potential need for further research on the effect of climate change conditions with longer dry spells during times of tillage operations^57,58^.

As already mentioned above, in our study, the differences between chisel and mouldboard plough are much higher compared to other studies (Table 2). However, normalising the literature values to an equal tillage depth of 0.25 m (using Eq. 2) leads to non-inversion tillage producing more tillage erosion (+ 42%; Table 2) compared to inversion tillage. This challenges the general idea of non-inversion tillage as a tool for soil conservation, which is only valid as long as tillage depth is substantially lower compared to inversion tillage. Currently, non-inversion tillage becomes more common in agricultural practices^59^ due to rising awareness of soils as a limited resource that drives an increasing implementation of soil conservation measures. Among many others, a major benefit of non-inversion minimal tillage is water and wind erosion reduction^4,6^ as remaining plant residues form protective soil cover^7^. This study demonstrates that non-inversion conservation tillage calls for substantially lower tillage depth to reduce tillage erosion. However, field sizes increased in developed countries globally over the last 60 years^60^, which fosters higher mechanisation that typically goes in hand with big farming structures for efficient, optimised cultivation^35,61^. Thereby, powerful machinery allow higher speed and depth of tillage operations, which is increasingly applied to non-inversion tillage practices due to the much lower energy and time demand (larger working width and possible tillage speed)^7,59,62^. However, the results of this study suggest a critical evaluation of the question if non-inversion tillage can serve as a soil protection measure against the background of individual agroecosystem conditions. It needs to be stressed that an application of non-inversion tillage with high speeds and high tillage depths cannot meet the goals of conservation tillage on rolling topography. In areas like Northeast Germany, where water erosion is about one order of magnitude lower than tillage erosion^12^ and non-inversion tillage is getting increasingly applied using big farming machines, the promotion of non-inversion tillage for soil conservation might result in large damage of precious soil systems.

## Conclusion

In this study we determined tillage erosion on paired plots to compare non-inversion chisel versus inversion mouldboard tillage while keeping tillage depth and speed constant. The results indicate that against most literature results, non-inversion tillage produces significantly more soil movement compared to inversion tillage. For the three tested slopes the translocation distance was by a factor of 1.3 to 2.1 larger in case of chisel tillage. The by far largest translocation distance and also *k*_*til*_ was found on the flattest slope, which showed low soil cohesion due to sandier and drier conditions during the experiment. This indicates an increasing climate sensitivity of tillage erosion in regions were dry soil conditions increase during spring season.

Our findings contradict the general assumption that non-inversion tillage reduces total erosion. This is supported by an analysis of standardised *k*_*til*_ values for different tillage implements of various studies. Especially in tillage erosion dominated areas with large-field farming using chisel tillage at high speeds and depths, calls for a critical evaluation if non-inversion tillage practices can still serve as soil conservation measure.

## Data Availability

The datasets generated and analysed during the current study are freely available and can be obtained from 10.13140/RG.2.2.24530.43203.
